# Polarizing T and B Cell Responses by APC-Targeted Subunit Vaccines

**DOI:** 10.3389/fimmu.2015.00367

**Published:** 2015-07-20

**Authors:** Gunnveig Grødeland, Even Fossum, Bjarne Bogen

**Affiliations:** ^1^Department of Clinical Medicine, K.G. Jebsen Centre for Influenza Vaccine Research (JIV), Oslo University Hospital, University of Oslo, Oslo, Norway; ^2^Centre for Immune Regulation (CIR), Institute of Immunology, University of Oslo, Oslo, Norway

**Keywords:** vaccine, APC targeting, T cells, antibody, Th1, Th2, influenza vaccines

## Abstract

Current influenza vaccines mostly aim at the induction of specific neutralizing antibodies. While antibodies are important for protection against a particular virus strain, T cells can recognize epitopes that will offer broader protection against influenza. We have previously developed a DNA vaccine format by which protein antigens can be targeted specifically to receptors on antigen presenting cells (APCs). The DNA-encoded vaccine proteins are homodimers, each chain consisting of a targeting unit, a dimerization unit, and an antigen. The strategy of targeting antigen to APCs greatly enhances immune responses as compared to non-targeted controls. Furthermore, targeting of antigen to different receptors on APCs can polarize the immune response to different arms of immunity. Here, we discuss how targeting of hemagglutinin to MHC class II molecules increases Th2 and IgG1 antibody responses, whereas targeting to chemokine receptors XCR1 or CCR1/3/5 increases Th1 and IgG2a responses, in addition to CD8^+^ T cell responses. We also discuss these results in relation to work published by others on APC-targeting. Differential targeting of APC surface molecules may allow the induction of tailor-made phenotypes of adaptive immune responses that are optimal for protection against various infectious agents, including influenza virus.

## Influenza and the Need for Novel Vaccines

Annual influenza epidemics are caused by antigenic drift, whereby mutations in the major surface proteins hemagglutinin (HA) and neuraminidase (NA) alter antigenic determinants. Consequently, vaccines against seasonal influenza have to be annually updated in order to match the circulating strains. On a more sporadic basis, new virions may form from reassortment, whereby antigenically different strains combine to form a new subtype. Such an antigenic shift could result in a new global pandemic. A wide selection of influenza A viruses continuously circulate in different species, making accurate predictions of reassortments and pandemics challenging. On this backdrop, it is important to develop vaccines that can offer broad protection against influenza, and that can be rapidly manufactured.

## Correlates of Protection

### Antibodies

About 80% of the proteins that protrude from the viral influenza membrane are HAs (1, 2). During infection, HA binds sialic acid residues on host cells to initiate virus–cell interactions and entry of the viral capsid into the cytosol. The immunodominant antigenic determinants on HA are mostly located in close proximity to the sialic acid binding receptor site, and represent mutation prone regions. Neutralizing antibodies against HA can block viral entry into host cells, and confer sterilizing immunity against influenza (3).

As induction of antibodies against HA is the focus of most current influenza vaccine strategies, several studies have shown that antibodies against NA may also be beneficial for clinical outcome (4–6). Although unable to block viral infection, antibodies against NA are thought to inhibit viral release from infected cells (7). In addition, antibodies against the extracellular domain of M2 have been shown to induce protection in animal models (8, 9). Whether anti-M2 antibodies are relevant in a human context remains unclear (10, 11).

### T cells

In addition to antibodies, an influenza infection triggers the development of virus-specific T cells. T cells can clear influenza infection in the absence of neutralizing antibodies (12, 13), and have in the elderly population been found a good correlate of protection (14). The ability to kill infected cells is mainly attributed to CD8^+^ T cells (15–17), and several of the CD8^+^ T cell subsets (Tc1, Tc2, Tc17) have independently been shown capable of mediating protection (18, 19). Typically, CD8^+^ cytotoxic T cells exert their function by secreting perforin, the polymerization of which forms a pore in the cell membrane that allows influx of serine proteases (20, 21), or by direct Fas–Fas ligand interactions (22, 23).

The main function of CD4^+^ T cells during influenza infections is to aid the development of cytotoxic T cells and antibodies (24, 25). The Th1 subtype of CD4^+^ T cells typically secrete interferon γ (IFNγ), and is associated with cellular immunity. However, Th1 cells can in addition help B cells, and IFNγ causes a switch to IgG2a. The hallmark cytokine of Th2 cells is interleukin 4 (IL4). Th2 cells are excellent helpers of B cells, and IL4 causes a switch to IgG1/IgE production (26). In accordance with the multiple functions of CD4^+^ T cells, it has been shown that mice lacking functional CD4^+^ T cells suffer more severe influenza infections, and that the development of immunological memory is impaired (27–29). In humans, pre-existing CD4^+^ T cells have been found to be associated with lower viral shedding (30), and in mice, a subset of CD4^+^ T cells that are able to directly lyse infected cells in a perforin-dependent manner have been described (31).

## Subunit Vaccines Against Influenza

Recently, a vaccine containing recombinant HA was licensed by the US FDA, thus laying the foundation for future vaccines containing recombinant influenza proteins (3). Subunit vaccines are considered safe, as they do not contain live viral components. However, a challenge of subunit vaccination is low immunogenicity. Several immunizations are typically needed for efficacy, and dose requirements are often high. These undesirable features have warranted the development of more potent delivery methods and adjuvants, which again could compromise the safety associated with subunit vaccination.

## Targeting of Antigen to APCs

The immunogenicity of subunit antigens can be increased by targeted delivery of antigen to antigen presenting cells (APC). In early studies, antigens were coupled to APC-specific antibodies by chemical conjugation (32–34), but genetic conjugations are now more common. Antigens can be linked directly to a Fab-fragment (35), included within loops of constant domains (36), or tail the C-terminus of the antibody heavy chain (37). In all these cases (32–37), the recombinant antibody-like molecules have APC-specific V-regions.

**Figure 1:**
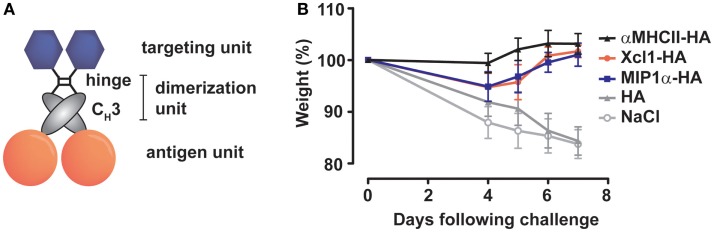
**Immunization with APC-targeted dimeric vaccines protect mice against influenza**. **(A)** The vaccine molecules consist of targeting units (scFv or natural ligands), dimerization units (hinge region and C_H_3 domain of human IgG3), and antigenic units [ex. influenza hemagglutinin (HA)]. **(B)** BALB/c mice were immunized with 25 μg DNA encoding the indicated vaccine molecules [HA from A/California/07/2009 (H1N1)] targeted toward MHC-II molecules (αMHCII-HA), chemokine receptors 1, 3, and 5 (MIP-1α-HA) or Xcr1 (Xcl1–HA). The mice were challenged 2 weeks after a single immunization with a lethal dose of influenza A/California/07/2009 (H1N1) and monitored for weight loss. All three APC-targeted vaccines induced protection against influenza, in contrast to vaccination with HA alone or NaCl. Modified with permission from Ref. (40).

We have previously generated novel vaccine molecules that were designed to mimic the bivalent receptor binding capacity of an antibody, display full-length antigens, and yet remain smaller than an Ig molecule. To achieve this, a single chain variable fragment (scFv) was linked to an antigen via the C_H_3-domain of human IgG3 (38). The C_H_3-domains will dimerize in the ER to generate bivalent display of antigens and scFvs (Figure 1A). Immunization with such vaccine molecules containing scFvs directed against major histocompatibility complex MHC class II (MHC-II) molecules, and expressing HA, have recently been shown to induce complete protection against influenza in immunized mice (39).

Natural ligands such as chemokines and toll like receptor (TLR) agonists may specifically bind receptors that are preferentially expressed on APCs. Thus, genetic fusion of antigen to natural ligands has been evaluated as a method to increase immunogenicity of subunit vaccination. A fusion between a tumor antigen and chemokine CXCL10 or CCL7 has been demonstrated to increase immune responses in immunized mice, and to protect against tumor challenge (41). Similar targeting approaches have been evaluated for influenza antigens, where the targeted delivery with chemokines such as CCL3 or XCL1, or the TLR ligand flagellin, have resulted in enhanced immunogenicity and protection against influenza (40, 42, 43).

Traditionally, the main rationale behind targeting of antigens to APCs has been to enhance antigen uptake and the subsequent presentation to CD4^+^ and CD8^+^ T cells. Dendritic cells (DC) are capable of efficient stimulation of both CD4^+^ and CD8^+^ T cells, and several APC targeting approaches have therefore focused on this population of cells. DC were first described in the 1970s (44), and are now generally divided into three sub-classes based on ontogeny as well as functional and transcriptional analysis (45). Plasmacytoid DC are highly efficient producers of type I interferon in response to TLR triggering, while conventional DC, cDC1 (Xcr1^+^Clec9a^+^), and cDC2 (CD11b^+^Sirp1a^+^) are the main stimulators of T cell responses. Both cDC1 and cDC2 are capable of presenting externally delivered antigen to CD4^+^ T cells, but cDC1 is considered superior at cross-presentation to CD8^+^ T cells (46, 47). Consequently, the specific targeting of antigen to cDC1 has gained attention as a method for induction of CD8^+^ T cell responses.

## Polarization of Immune Responses

In a recent series of papers, we have evaluated the efficacy of a single immunization with influenza HA targeted to MHC-II molecules, chemokine receptors (CCR) 1, 3, and 5, or Xcr1 (39, 40, 42). For targeting of MHC-II molecules, HA was fused, via a dimerization domain, to a scFv specific for murine I-E^d^ (αMHCII-HA). Similarly, targeting to CCR1/3/5 and Xcr1 was performed by fusing HA to the chemokines MIP-1α (MIP1α-HA) or Xcl1 (Xcl1–HA), respectively. MHC-II molecules are expressed on all professional APC, including B cells, macrophages (MΦ), and DC. CCR1/3/5 are expressed on MΦ, DC, eosinophils, and T cells, while Xcr1 is selectively expressed on cDC1 (48, 49). All three targeting approaches induced HA-specific immune responses, and protected mice against a lethal challenge with influenza virus (Figure 1B), in contrast to non-targeted controls (39, 40, 42).

While conferring protection against influenza, targeted delivery of HA to MHC-II molecules, CCR1/3/5, or Xcr1 revealed qualitative differences in induced immune responses. Targeting of HA to MHC-II molecules induced a Th2 dominant response characterized by IL4-secreting CD4^+^ T cells, although some IFNγ^+^ T cells were also observed (39, 42). MIP1α-HA induced higher numbers of IFNγ-secreting cells, and was found to polarize the immune response toward Th1 cells (42). In an assessment of T cell contribution to protection, depletion of CD8^+^ T cells in mice previously immunized with MIP1α-HA abolished protection against influenza. By contrast, depletion of CD8^+^ and CD4^+^ T cells after immunization with αMHCII-HA did not diminish the induced protection (42). The importance of antibodies after immunization with αMHCII-HA was confirmed by the early presence of neutralizing antibodies in sera, and ultimately by the demonstration that transfer of sera from immunized mice could protect naïve mice against a lethal influenza challenge (39). It was also shown that the fairly low amounts of T cells induced could confer protection against influenza in the absence of relevant antibodies (39). Thus, immunization with MIP1α-HA induces CD8^+^ T cell mediated protection, while αMHCII-HA induces neutralizing antibodies and T cells that probably act in concert.

MIP1α-HA targets several cell populations, whereas Xcl1-targeted vaccines have been demonstrated to specifically bind Xcr1 expressed on cDC1 (40). Adoptive transfer of OT-I and OT-II cells to Xcr1^−/−^ knockout or wild type mice, prior to immunization with Xcl1–OVA, demonstrated that efficient proliferation was dependent upon functional targeting of antigen to Xcr1 (40). Similar observations have been made for Xcl1–OVA delivered by laser-assisted intradermal delivery (50) or for OVA directly fused to Xcl1 or to an antibody specific for Xcr1 (51). Direct conjugation of antigen to Xcl1 was required for efficacy, since delivery of unconjugated Xcl1 together with OVA failed to enhance proliferation (50). The importance of a direct conjugation has also previously been demonstrated for antigen linked to the chemokine MIP3α (52), and for a T cell epitope linked to CD40-specific V regions (53). Together, these results indicate that the observed immune responses are associated with receptor uptake, rather than a chemokine induced adjuvant effect.

Similar to MIP1α-HA, vaccination with Xcl1–HA as DNA induced a Th1 type of immunity, characterized by a marked increase of IFNγ-secreting CD4^+^ T cells (40). Correspondingly, Xcl1–HA induced cytotoxic CD8^+^ T cells that killed target cells presenting HA-derived peptides on MHC class I molecules. Depletion of Xcl1–HA-induced CD8^+^ T cells before viral challenge also confirmed that these cells played a central role in mediating protection against influenza (40). Immunization with Xcl1–OVA protein also resulted in the induction of cytotoxic CD8^+^ T cell responses when administered i.v. in combination with LPS (51). Interestingly, laser-assisted intradermal delivery of Xcl1–OVA protein induced enhanced cytotoxic CD8^+^ T cell responses in the absence of adjuvant (50). In both these studies, targeting of OVA to the Xcr1 receptor induced protection in a murine melanoma tumor model (50, 51). Taken together, these three studies (40, 50, 51) highlight Xcr1 as a potent target for the induction of cytotoxic CD8^+^ T cells.

**Figure 2:**
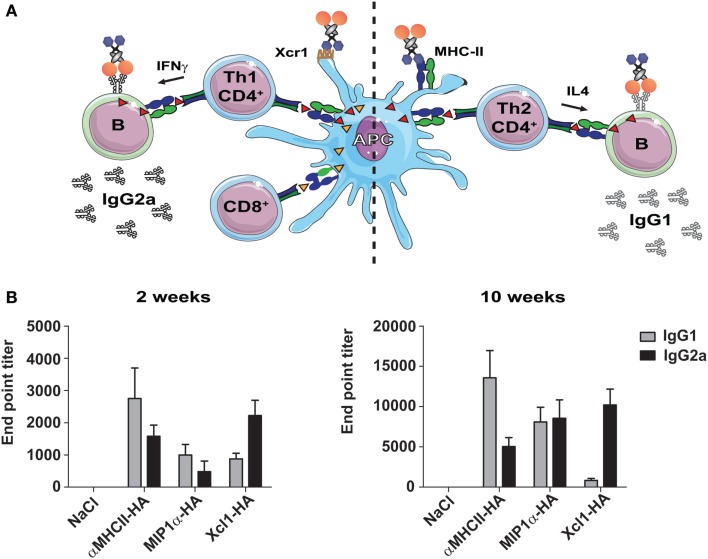
**Targeting of selected surface receptors on APC will influence the vaccine-induced Th1/Th2 polarization and antibody subtypes**. **(A)** Illustration of the different immune responses as induced by targeting of antigen to the chemokine receptor Xcr1 (left side) or MHC-II (right side). *Left side*: Targeting of antigen to Xcr1 induces IFNγ-secreting CD4^+^ Th1 cells that can provide help to B cells and promote the formation of IgG2a antibodies. In addition, targeting of Xcr1 results in presentation of peptides on MHC-I molecules, and induction of strong CD8^+^ T cell responses. *Right side*: Targeting of antigen to MHC-II molecules induces CD4^+^ Th2 cells that secrete IL4, and that can provide help to B cells and promote the formation of IgG1 antibodies. **(B)** BALB/c mice were immunized with 25 μg DNA encoding the indicated vaccine molecules [HA from influenza A/Puerto Rico/8/1934 (H1N1)], and serums samples were harvested 2 or 10 weeks after a single immunization. Serum levels of HA-specific IgG1 and IgG2a antibodies were determined by ELISA against inactivated influenza A/Puerto Rico/8/1934 (H1N1). Modified with permission from Ref. (40).

Different types of immunity are associated with the induction of different IgG subclasses. When CD4^+^ T cells provide help to B cells, they also directly influence isotype switching. IFNγ secreted by CD4^+^ Th1 cells will promote the secretion of IgG2a, whereas IL4-secreting Th2 cells promote switching to IgG1 (54). Consequently, an assessment of IgG1 vs IgG2a could indicate the degree of induced Th1/Th2 immune polarization (Figure 2A). As earlier mentioned, HA targeted to MHC-II molecules induced higher levels of IL4 secreting CD4^+^ T cells, and strong antibody responses. While such targeting increased responses for most IgG subclasses, IgG1 was indeed dominant (39) (Figure 2A). Targeting of HA to Xcr1 also resulted in increased antibody responses as compared to non-targeted controls, but these were predominantly IgG2a (40) (Figure 2B). In contrast to Xcl1–HA, MIP1α-HA induced a lower and more mixed humoral response, with both IgG1 and IgG2a being present (42). It is likely that the selective targeting of cDC1 cells caused a more stringently Th1-polarized immune responses observed after vaccination with Xcl1–HA, as opposed to the more mixed responses observed after vaccination with MIP1α-HA. In summary, the three targeting approaches induce different types of humoral responses, with MHC-II-targeting promoting Th2/IgG1, CCR1,3,5-targeting giving a mixed IgG1/IgG2a response, and Xcr1-targeting polarizing responses toward Th1/IgG2a (Figure 2B).

Interestingly, these observations suggest that the choice of APC receptor may be used to direct immune responses toward particular antibody subclasses. This is of importance since the different IgG subclasses vary in their ability to induce antibody dependent cell-mediated cytotoxicity (ADCC) and complement activation, partly through different affinities for FcγR [reviewed in Ref. (55)]. While IgG2a binds strongly to activating FcγR, such as FcγRI, III, and IV, IgG1 have higher affinity for the inhibitory FcγRIIb receptor (56). Consequently, IgG2a antibodies induce stronger ADCC and complement activation than IgG1. Interestingly, two recent studies have suggested that broadly neutralizing antibodies against both HIV and influenza mediate their effect through FcγR binding, and that antibodies of the IgG2a subclass therefore are more efficient at this than IgG1 (57, 58). By contrast, strain-specific neutralizing antibodies against HA do not require FcγR binding, and function equally well as both IgG1 and IgG2a (58). Since IgG2a antibodies induce stronger ADCC, they might also be associated with an increased risk of induced cytopathic effects to host cells (59). Thus, in situations where FcγR binding is not required for induction of protection, it may be beneficial to induce IgG1. All this considered, our results would suggest to target antigens to MHC-II molecules for induction of specific neutralizing anti-HA antibodies, whereas targeting to Xcr1 would be more beneficial for induction of broadly neutralizing antibodies against conserved HA epitopes, such as the stem.

## Immune Polarization: A Function of Targeted Receptor or the APC that EXPRESS the Particular Receptor?

Since the targeted receptors are differently distributed on various cell types, a relevant question is whether the targeted cell type will determine the observed polarizations. Some studies have focused on antibody-mediated targeting of Clec9A for vaccinations (60–62). With its selective expression on cDC1 cells, the Clec9A-targeted approach is comparable to Xcl1–mediated delivery of antigen to Xcr1. Targeting of antigen to Clec9A has been reported to enhance proliferation of antigen-specific CD4^+^ and CD8^+^ T cells, and to confer protection against melanoma (60, 63). These observations correlate with our results in that Xcl1–HA induced T cell-mediated protection against influenza. In addition, targeting of Clec9A induced strong antibody responses, with an efficient induction of T follicular helper cells (T_FH_) (61, 62). Although the molecular mechanisms for how Clec9A induce antibodies are not known, T_FH_ cells are presumably a key in that they are important for germinal center (GC) formation and induction of antibody secreting plasma cells (62). Interestingly, targeting of antigen to Clec9a was reported to induce more IgG1 than IgG2a (64), suggesting the induction of a more Th2 polarized CD4^+^ T cell response. Together, this may suggest that it is the targeted receptor, rather than the targeted APC type, that determine the outcome of the immune response in these examples (40).

It should be noted that the studies targeting Clec9A and Xcr1 were done using different immunization protocols and different mouse strains, raising the possibility that other factors have influenced the results. However, experiments with targeting of Clec9A showed Th2-like responses in Th1-prone C57BL/6J mice, and targeting of antigen to Xcr1 induced Th1-polarization in both Th2-prone BALB/c mice (40) and Th1-prone C57BL/6J mice (50, 51). Similarly, the Th2 polarization observed after targeting of HA to MHC class II molecules has been confirmed in both BALB/c and Th1-prone B10.D2 mice (39).

## Receptor Expression and Endocytosis

The expression level of surface receptors and endocytosis rates could play a major role in determining the efficacy by which targeted vaccination stimulates presentation of peptides on MHC-I/II to T cells. In a recent study, comparing targeting of antigen to DEC205, Clec9a, CD11c, CD11b, and CD40, it was shown that delivery to DEC205 resulted in about 80% of surface receptors being internalized by 90 min. By contrast, delivery to CD11c or CD11b internalized surface receptors more slowly and inefficient (65). The authors concluded that endosomal trafficking of endocytosed antigen was likely to influence the efficacy of antigen presentation, a factor which has previously been suggested to influence cross-presentation to CD8^+^ T cells (66, 67). While endocytosis is necessary for presentation of peptides from internalized antigens to MHCI/II molecules, and thus also activation of T cells, it is possible that reduced endocytosis might favor the stimulation of B cells and antibody production by allowing an extended period of time where B cells can recognize surface antigens.

## Efficient Induction of Humoral Immune Responses

Targeting of antigen to MHC-II molecules was shown early on to increase serum responses in the absence of adjuvant (68). In addition to the above mentioned studies targeting Clec9A, other groups have identified CD11c and CD180 as particular interesting receptors for induction of strong antibody responses (69–71). CD11c is predominantly expressed on DC, with more minor expression on monocytes, MΦ, neutrophils, and some B cells. CD180 is expressed on B cells, DC, and MΦ.

Ligation of CD180 on B cells has been reported to induce activation and proliferation, and may explain why targeting of antigen to CD180 could activate CD4^+^ T cell-independent IgG responses after immunizations of CD40-KO and TCR α/δ KO mice (71–74). This is in contrast to our experiments with targeting of HA to MHC-II molecules, since immunization of thymus-deficient mice indicated that the humoral responses were T cell dependent (39). The mechanism behind the strong antibody responses induced by αMHCII-HA remains to be elucidated, but the rapid formation of IgG in sera (day 8 after a single vaccination) points toward rapid affinity maturation and GC formation. It is conceivable that the responses in this respect are aided by the vaccine molecules forming an APC-B cell synapsis (75, 76), where the bivalent vaccine molecules bridge MHC class II molecules on APCs and antigen-specific B cell receptors on B cells.

## Conclusion

We have here discussed how proper selection of target receptors on APC may polarize immune responses toward either dominant cellular or antibody responses. Furthermore, the immune responses could be tailor-made with respect to IgG isotypes and Th1/Th2 dominance. Given the importance of neutralizing antibodies in protection against influenza, targeting of antigen to MHC class II molecules should be further evaluated in larger mammals and humans. While antibodies against the influenza virus surface proteins are important, T cell responses against the conserved internal influenza antigens could offer broader protection. For eliciting strong T cell responses, use of vaccine antigens that are targeted by use of chemokines MIP-1α and Xcl1 could be important. In the future, more APC targets for vaccines should be tested for their ability to influence magnitude and polarization of immune responses. Also, a deeper understanding of the relationship between APC target specificity and immune response polarization is needed.

## Conflict of Interest Statement

Gunnveig Grødeland, Even Fossum, and Bjarne Bogen are inventors on patent applications filed on the vaccine molecules by the TTO offices of the University of Oslo and Oslo University Hospital, according to institutional rules. Bjarne Bogen is head of the Scientific panel in Vaccibody AS.
